# Impact of Ph.D. training: a comprehensive analysis based on a Japanese national doctoral survey

**DOI:** 10.1007/s11192-017-2479-7

**Published:** 2017-08-11

**Authors:** Sotaro Shibayama, Yoshie Kobayashi

**Affiliations:** 1 Sten K. Johnson Centre for Entrepreneurship, School of Economics and Management, Lund University, P. O. Box 7080, 220 07 Lund, Sweden; 2 National Institute of Science and Technology Policy, Ministry of Education, Culture, Sports, Science and Technology, Tokyo, Japan; 3 The Graduate School of Pharmaceutical Sciences, University of Tokyo, Tokyo, Japan

**Keywords:** Ph.D. training, Academic training, Higher education, Postgraduate education, Academic career

## Abstract

Ph.D. training in academic labs offers the foundation for the production of knowledge workers, indispensable for the modern knowledge-based society. Nonetheless, our understanding on Ph.D. training has been insufficient due to limited access to the inside of academic labs. Furthermore, early careers of Ph.D. graduates are often difficult to follow, which makes the evaluation of training effects challenging. To address these limitations, this study aims to illustrate the settings of Ph.D. training in academic labs and examine their impact on several training outcomes, drawing on a national survey of a cohort of 5000 Ph.D. graduates from Japanese universities. The result suggests that a supervising team structure as well as the frequency of supervision, contingent to a few contextual factors, determine the Ph.D. graduates’ career decisions, performance, and degrees of satisfaction with the training programs.

## Introduction

The modern society is increasingly becoming knowledge-driven and major challenges our society faces today require solutions with scientific expertise, and thus, the development of human capital at the knowledge frontier is crucial for the sustainability of our society (Bozeman et al. 2001). The development of knowledge workers typically takes the form of postgraduate education, in which research training (*academic training*, hereafter) plays an essential role. Academic training is a significant investment that costs students several years or possibly longer and supervisor’s considerable time and efforts (Stephan 2012). Nevertheless, the contemporary academic training practices have been criticized, for example, for failing to meet changing societal needs and for producing excessive Ph.D.s (National Research Council 1998; Cyranoski et al. 2011).

These problems in academic training are partly attributable to a gap between (mass) education policies and science policies. Further, recent policies have stressed accountability that is often translated into short-term and merit-based evaluation, and a relatively long-term payoff from academic training tends to be overlooked (Hackett 1990). A similar gap exists in literature between studies on higher education and those on knowledge production. Though academic career design has been a popular subject (e.g., Allison and Long 1990; Geuna 2015; Stephan 2012), early careers are relatively understudied. Among others, empirical difficulty in accessing two types of data has been compromising our understanding on academic training. First, prior studies had poor access to the inside of academic labs where training takes place. Ethnographies in sociology of science have illustrated the details of lab operation (Campbell 2003; Delamont and Atkinson 2001; Delamont et al. 1997; Salonius 2007), but their implications are restricted to certain lab contexts. Second, tracing early careers of academics is often challenging. A few countries have implemented surveys to follow the careers of Ph.D. graduates; such as Science and Engineers Statistical Data System (SESTAT) in the USA and Destinations of Leavers from Higher Education (DLHE) in the UK. These systematic efforts have contributed to our understanding on early careers of academics (Agarwal and Ohyama 2012; Roach and Sauermann 2010). Nonetheless, career data and training data have rarely been integrated, and thus, we still have insufficient understanding on how academic lab training leads to the development of S&T human capital.

The objective of this study is to address these gaps with the national survey of Japanese Ph.D. graduates, which inquired into both Ph.D. training settings and traced their careers. The population of the survey is a cohort of Ph.D. students who graduated from Japanese universities in 2012, and 5052 responses were collected in 2014. The result finds that supervisory settings—a supervising team and frequency of supervision—influence the Ph.D.s’ career decisions, scientific and economic performance, and their level of satisfaction on the Ph.D. program.

The remainder of this article is organized as follows. “Literature review” section reviews previous literature. “Context of academic training in Japan” section overviews the Japanese postgraduate education system. “Data and methods” section explains our data. “Results” section presents the results. “Discussions” section discusses the results and implications.

## Literature review

### Linking lab training and training outcome

Postgraduate education programs employ various education approaches, usually involving (1) a general component that provides students with knowledge commonly needed across the discipline, often through mass teaching, and (2) a specific component that aims to develop knowledge and skills concerning a certain area of expertise specific to the lab through a research project, or *academic training*. Prior literature on higher education has paid relatively limited attention to the latter compared to the former. This is partly because of empirical difficulty in observing the inside of labs, where academic training occurs. A lab consists of a team of scientists including a supervising professor and junior members including students (Delamont and Atkinson 2001; Latour and Woolgar 1979; Owen-Smith 2001). The core part of academic training employs the apprenticeship model, where students are tasked to solve research questions as a member of a research project under the supervision of professors (National Research Council 1998). Some anthropological studies did investigate the inside of academic labs in depth, illustrating how academic science operates in specific labs in a great detail (Delamont and Atkinson 2001; Delamont et al. 1997; Knorr-Cetina 1999; Latour and Woolgar 1979; Salonius 2007), but academic training was not necessarily of their primary interest and a general picture is lacking (Shibayama et al. 2015). Scientometric techniques have been developed to identity student-supervisor relationships (Lariviere 2012; Morichika and Shibayama 2016), but they cannot reveal the details of the interpersonal relationships.

Tracing postgraduate careers of students presents another challenge. While identifying established academics is fairly feasible thanks to increasingly available career data of academics (Gaughan and Bozeman 2002), early careers are still difficult to identify because academic jobs in early stages tend to change frequently and be made insufficiently public. Moreover, if graduates are employed outside academia, their career information is usually kept private, and even if it is publicly available, linking it with education record poses another challenge. Addressing these difficulties require systematic and perhaps authoritative efforts for data collection. Indeed, a few national surveys have been implemented, such as SESTAT in the USA and DLHE in the UK, and contributed to our understanding on higher education systems. For example, Agarwal and Ohyama (2012) used SESTAT to investigate the link between scientists’ ability, preferences, and their career development. Roach and Sauermann (2010), drawing on Survey of Doctorate Recipients (SDR) in the USA, predicted the innovative performance of Ph.D. graduates based on their motives. Nevertheless, the focus of these surveys is to follow postgraduate careers rather than to understand pre-graduate conditions. To link the two elements, therefore, scholars have needed to rely either on additional data sources or on their original surveys in smaller scales.

### Training outcomes

The current study aims to address these issues by investigating the impact of academic training on three aspects of training outcome: Ph.D. students’ (1) performance, (2) career choice, and (3) subjective evaluation on training programs. In what follows, we discuss the rationale of these outcome aspects in connection with previous literature.

First, students’ subjective evaluation is of practical use for the evaluation of Ph.D. programs, since it is relatively easy to measure without following students’ postgraduate careers. Previous literature is rather developed in this area. The higher education literature evaluated Ph.D. programs from the perspective of students in various dimensions (e.g., Hockey 1996; Kam 1997; Marsh et al. 2002). For example, Morrison et al. (2011), drawing on a survey of Ph.D. graduates in Social Sciences in the USA, found that the quality of advice from dissertation supervisors is associated with students’ evaluation on the excellence of Ph.D. programs. Similarly, Mainhard et al. (2009) suggested that the availability of Ph.D. supervisors is a key determinant of the perceived quality of Ph.D. supervision. These studies have confirmed that lab settings and the interpersonal relationship between students and supervisors play a critical role in academic training. Nonetheless, they have had limited link with more objective evaluation such as performance or with longer-term outcome such as career development. This is why we incorporate two other outcome aspects.

Second, students’ career choice is also of practical interest as Ph.D. graduates are taking increasingly diverse types of jobs. As above mentioned, however, a theoretical link between higher education and later career development has been insufficient. The academic career is a traditional and increasingly popular research subject (e.g., Agarwal and Ohyama 2012; Geuna 2015; Long et al. 1979). For example, many studies found that the prestige of degree-awarding departments determines the destination of academic careers (e.g., Baldi 1995; Crane 1965; Debackere and Rappa 1995). Long et al. (1979) analyzed postgraduate careers of biochemical Ph.D.s in the US and found that the prestige of the first academic jobs is significantly influenced by the performance of Ph.D. supervisors in addition to the prestige of the degree-awarding departments. Nonetheless, the literature has rarely examined the detail of supervisory settings, relying on easily observable factors. Thus, this study aims to contribute to the literature by investigating the detailed operation of academic training.

Third, students’ performance is of obvious relevance, on which the effectiveness of academic training should be evaluated. As such, performance itself has been widely studied, and in particular, the link between higher education and performance outcome has been studied to some extent. For example, the literature in sociology and in science policies has found the organizational prestige and supervisors’ performance to be strong predictors of students’ postgraduate performance (Allison and Long 1990; Geuna 2015; Long and McGinnis 1985). A line of literature on the organizational design of labs, either in industry or in academia, has also been investigating various organizational factors such as prestige, age, and size as determinants of performance (e.g., Heinze et al. 2009; Pelz and Andrews 1966). Again, however, the literature lacks for depth in the operation of training with few exceptions (Shibayama et al. 2015).

### Supervisory settings

As to the operation of academic training, practices inside labs have been studied in the higher education and education psychology literature. Since academic training is a highly personal process and shaped by the interaction between individual supervisors and students (Brown and Atkins 1988; Hockey 1991), the literature scrutinizes variations in training styles and motives among individual supervisors. For example, Hockey (1996) identified sets of motives behind academic training, such as the need for students’ labor and moral obligation for education. Murphy et al. (2007) also found “controlling” and “guiding” beliefs as supervisors’ distinctive roles in academic training.

Ethnographies in sociology of science have detailed the daily operation of lab research (Delamont and Atkinson 2001; Delamont et al. 1997; Knorr-Cetina 1999; Latour and Woolgar 1979; Salonius 2007). In particular, they illustrated the allocation of research-related tasks between students and supervisors. A stylized view is that supervisors are responsible for upstream tasks such as problem identification and coordination while students engage in downstream tasks such as experiments (Laudel 2001; Traweek 1988). For example, Delamont et al. (1997), using a sample of British universities, found that supervisors are responsible for identifying research projects and assigning them to students, while students typically consider their lab experience as an opportunity to acquire technical skills.

This study also investigates the details of supervisory settings, particularly, in terms of the intensity of supervision and team structure. The intensity, or the frequency, of supervision has been relatively well studied (Hockey 1991). For example, Wright and Lodwick (1989) suggested that frequent supervision increases the likelihood of successful degree attainment. The intensity of supervision is of practical relevance, since the current higher education policies tend to give more emphasis to research than to education (Cyranoski et al. 2011; Gould 2015), where training efforts could be replaced by research efforts. Recent policies in many countries also tend to produce an increasing number of postgraduate degrees (Cyranoski et al. 2011), which can reduce training effort for each student (Salonius 2007) and lower the quality of training outcome (Shibayama and Baba 2015).

To investigate these issues in depth, the current study also incorporates the structure of supervising teams. As the complexity of science has been increasing, research projects need more interdisciplinary collaboration (Wuchty et al. 2007). This applies to academic training (Spelt et al. 2009), where teams of student supervision and evaluation tend to include multiple members with various backgrounds. Nonetheless, this aspect has been understudied possibly due to the assumption that students have a single supervisor in the traditional apprenticeship model. The structure of a supervising team is interrelated with the intensity of supervision, because efforts for training is shared among multiple instructors in a team. For example, as later described in this study, a busy professor can delegate his training role to postdocs in the same lab. Even if their supervision is frequent, the training effect should be questionable given their limited experience.

## Context of academic training in Japan

In Japan, approximately 700 universities offer 4-year undergraduate programs, among which approximately 400 universities offer Ph.D. programs. They are grouped into three types based on governing bodies: national, regional (of prefectures or cities), and private. Among the three, national universities are the main player of scientific research and academic training while most private universities focus on undergraduate education. For example, national universities accounted for 75% of 12,000 Ph.D. degrees while private universities accounted for 77% of 564,000 bachelor degrees awarded in 2014.^1^

Most postgraduate education programs in Japan consist of a 2-year master program and a 3-year Ph.D. program.^2^ A majority of graduate students decide whether to proceed to a Ph.D. program during a master program (Kato et al. 2012). Once students are admitted to Ph.D. programs, drop out is rare, and students graduate with limited delay. For example, 50% of the students who enrolled in Science and Engineering Ph.D. programs in 2008 graduated in 3 years, 79% within 4 years (plus 1 year), and 91% within 6 years (plus 3 years). Graduation in the Japanese Ph.D. system does not necessarily mean that students have successfully earned degrees. Students can choose to graduate Ph.D. programs as long as they meet certain credit conditions, and after graduation they can apply for degrees as soon as completing dissertations.^3^ In fact, 22% of Ph.D. graduates in our sample graduated without a degree. This is more common in Humanities, Arts, and Social Sciences (HASS) than in Science, Technology, Engineering, and Mathematics (STEM).

In most Ph.D. programs, each Ph.D. student is officially under the supervision of a single professor. In practice, however, there is a significant variation in the supervisory settings. The variation is attributed to a few sources, including the setting of the official supervisor’s lab and the policies or the environment of the department that offers the Ph.D. programs. As for the latter, multiple faculty members in the same department usually participate in the dissertation evaluation committee, and they sometimes play a proactive role in supporting Ph.D.s from early program stages. As for the former, a lab usually involves other students and staff, who can also participate in the supervision of students. Particularly, national universities in STEM fields tend to adopt so-called chair system modelled on the German system, where a senior professor organizes a lab and supervises not only students but also junior professors. In this hierarchical structure, the supervision of students is often in part or whole delegated to junior professors, postdocs, and even senior students. The chair system sometimes causes organizational barriers between labs, restricting students’ interaction with researchers in other labs.

Ph.D. programs in Japan used to be mainly meant to train academic researchers, so most students enrolling in Ph.D. programs pursued academic careers. However, around the 1980s and 1990s, the postgraduate education system was repositioned for the training of knowledge workers in general to satisfy diversifying societal needs (Ehara and Umakoshi 2004: Ch. 3). A series of system reform increased the admission quota for postgraduate programs, and many postgraduate programs were newly opened.^4^ It also allowed candidates who already have jobs to enroll in Ph.D. programs and pursue degrees often in part-time without quitting the jobs. This so-called “professional” Ph.D. has become common in applied fields such as Medicine and in Social Sciences. Recent years have also seen an increasing number of international Ph.D.s. Overall, the number of Ph.D. students was doubled in 1991–2000. The rapid expansion of the postgraduate system, however, has been heavily criticized for compromising the quality of Ph.D. training. In addition, employment conditions for recent Ph.D. graduates are often unstable (Cyranoski et al. 2011).^5^ Consequently, academic careers have become a less popular option for students, which partially contributed to a recent decline in Ph.D. enrolment (Morichika and Shibayama 2016).

## Data and methods

### Survey data

This study draws on a national survey, Japan Doctoral Human Resource Profiling (JD-Pro). The population of JD-Pro was the entire cohort of 16,445 Ph.D. students who graduated from Ph.D. programs in Japanese universities in the academic year of 2012. It covered all disciplines and all Japanese universities that offer Ph.D. programs. The survey was carried out in 2014, 1.5 years after their graduation. JD-Pro included several sets of questions concerning Ph.D. training programs, employment after graduation, research activities, and so forth. This study particularly draws on the questions about supervisory settings for Ph.D. training and several outcome measures. The survey was conducted both on a web-based system and by mail and collected 5052 effective responses (response rate = 38.1%). Kobayashi (2015) reports the detail of the survey. The sample consists of international Ph.D.s (15%), professional Ph.D.s (34%), and regular Ph.D.s (52%) in the fields of Science (17%), Engineering (24%), Agriculture (7%), Health (29%), Humanities (8%), Social sciences (9%), and others (6%). The mean age is 38, and 28% are female.

### Measures

#### Supervisory setting

The survey had a section of questions regarding supervisory settings. In particular, it asked about two main researchers who most frequently gave instructions in research projects, among the official supervisor, internal faculty members (i.e., in the same university) other than the official supervisor, external faculty members (i.e., in different universities), and non-faculty researchers (typically, senior students or postdocs in the same lab). It subsequently inquired into the frequency of instruction given by the two researchers. Based on these measurements, we prepared two sets of variables. The first set is the frequency of instruction given by the four categories of researchers: (1) the official supervisor (*Official supervisor*), (2) internal faculty members (*Internal faculty*), (3) external faculty members (*External faculty*), and (4) non-faculty researchers (*Non*-*faculty*). Each variable takes a five-point scale, 0: never, 1: once a half year or less, 2: once a quarter, 3: once or twice a month, 4: once a week or more. The second set is a single variable, the number of faculty members (i.e., excluding non-faculty researchers) engaged in Ph.D. instruction once a month or more frequently (#*Faculty*). The variable takes a value of 0, 1, or 2.^6^

#### Outcome of Ph.D. training

This study draws on three sets of outcome variables. The first set consists of three variables concerned with Ph.D.s’ postgraduate careers. First, we study the choice between academic and non-academic careers. The survey inquired into several questions on the employment conditions of the respondents at the time of the survey.^7^ We coded a dummy variable 1 if a respondent had a job in an academic organization (i.e., a university or a public research organization) and 0 otherwise (e.g., in a private company) (*Academic career*). Second, we test whether students obtained a degree in time because degree attainment is likely to influence their career decisions. We coded a dummy variable 1 if a degree was awarded within the standard time period and 0 otherwise (*Degree in time*). Third, we examine the relationship between the job and the subject of Ph.D. dissertations to evaluate whether the knowledge learned in Ph.D. programs are used in postgraduate careers. We coded a dummy variable 1 if a respondent’s job is related to his or her Ph.D. dissertation and 0 otherwise (*Related job*).

The second set consists of two variables concerned with performance. Because the majority of Ph.D. graduates are engaged in research jobs, we draw on scientific publication as a performance measure. For those who had research jobs, we counted scientific articles they published before the time of the survey (*#Pub*). While most Ph.D.s who obtained jobs in academia continued research, only 56% of those at non-academic jobs were engaged in research. To address this limitation for non-academic workers, we also measured the wage rate as a proxy of performance (*Wage rate*).

The final set of outcome variable consists of a single measure based on the subjective evaluation by the respondents. Namely, we examine Ph.D. students’ satisfaction with the program in a five-point scale ranging from 1: not satisfied to 5: satisfied (*Ph.D. satisfaction*).

#### Control variables

The regression analyses control for several factors. We include three dummy variables corresponding to the student types (*regular Ph.D., professional Ph.D.,* and *international Ph.D.*) and seven dummy variables for Ph.D. fields (*Ph.D.s in Science, Engineering, Agriculture, Health, Humanity, Social Sci,* and *Others*). As a proxy of the performance of supervisors, we control for the prestige of degree-awarding universities. For this, we grouped Japanese universities into four tiers on the basis of publication shares at the university level and coded the top tier 4 and the bottom tier 1 (*Univ tier*).^8^

We also include several control variables for individual attributes. We control for the age (*Age*) and gender (*Female*) of the respondents. To proxy respondents’ performance prior to Ph.D. training, we include a dummy variable coded 1 if a respondent had a national Ph.D. fellowship that is awarded on the basis of their performance before the Ph.D. course (*Fellowship*).^9^ We also control for reasons why the respondents decided to pursue Ph.D. degrees. In particular, we include a dummy variable coded 1 if the motive was “to become an academic teacher or researcher” (*Academic motive*) and another dummy variable coded 1 if the motive was “to delay job hunting” (*Job motive*).

## Results

Table 1 presents the descriptive statistics and correlation matrix of the variables. Concerning the career outcomes, 51% of the respondents were awarded Ph.D. degrees in time; 57% chose academic careers; 89% had jobs somewhat related to their Ph.D. dissertations. The median count of publications is three, and the average wage rate is 2200 JPY per hour. 80% of Ph.D.s were satisfied with the training they received.

**Table 1:** Descriptive statistics and correlation matrix

Variables	Mean	SD.	Min	Max	1	2	3	4	5	6	7	8	9	10	11
1 Degree in time	.510	.500	.000	1.000											
2 Academic career	.574	.495	.000	1.000	.002										
3 Related job	.894	.308	.000	1.000	.046	.228									
4 ln(#Pub)	1.334	.654	.000	3.951	.166	.108	.050								
5 Wage rate	2.214	1.576	.000	17.308	−.034	−.211	−.011	.031							
6 Ph.D. satisfaction	4.116	1.025	1.000	5.000	.109	.049	.107	.113	.002						
7 Age	38.338	8.494	27.000	67.000	−.202	−.134	−.030	−.022	.385	.042					
8 Female	.276	.447	.000	1.000	−.111	.099	−.019	−.069	−.088	−.014	.064				
9 Fellowship	.067	.250	.000	1.000	.126	.054	.031	.061	−.100	.014	−.190	−.049			
10 Job motive	.039	.194	.000	1.000	.032	−.043	−.061	−.046	−.077	−.040	−.127	−.031	.016		
11 Academic motive	.374	.484	.000	1.000	−.019	.277	.056	.083	−.223	.004	−.121	.014	.080	−.031	
12 Regular Ph.D.	.515	.500	.000	1.000	.048	.076	−.006	−.084	−.263	−.099	−.462	−.051	.203	.110	.080
13 Professional Ph.D.	.338	.473	.000	1.000	−.052	−.154	−.017	.044	.479	.024	.552	−.019	−.189	−.130	−.201
14 International Ph.D.	.146	.353	.000	1.000	.001	.098	.032	.060	−.287	.109	−.086	.097	−.034	.018	.156
15 Ph.D. in Science	.175	.380	.000	1.000	.032	.015	−.034	−.030	−.149	−.012	−.213	−.078	.159	.024	.034
16 Ph.D. in Engineering	.235	.424	.000	1.000	.127	−.134	.008	.115	−.024	.085	−.026	−.194	−.010	.065	−.052
17 Ph.D. in Agriculture	.065	.247	.000	1.000	.051	−.008	−.008	.041	−.069	−.006	−.026	−.013	.042	.025	−.014
18 Ph.D. in Health	.289	.454	.000	1.000	.152	.054	.024	−.027	.251	−.074	.061	.081	−.094	−.055	−.119
19 Ph.D. in Humanity	.083	.277	.000	1.000	−.254	.024	−.004	−.036	−.097	−.006	.068	.125	−.032	−.028	.095
20 Ph.D. in Social Sci	.090	.287	.000	1.000	−.178	.021	−.009	−.084	.009	.021	.123	.022	−.038	−.020	.074
21 Ph.D. in Others	.062	.241	.000	1.000	−.110	.066	.015	−.002	−.031	−.004	.069	.155	−.016	−.018	.079
22 Univ tier	2.267	1.162	1.000	4.000	.084	.035	.033	.054	−.066	−.019	−.194	−.108	.221	.051	.068
23 Official supervisor	3.180	1.135	.000	4.000	.091	.030	.046	.004	−.099	.337	−.128	−.033	.019	.037	.024
24 Internal faculty	1.509	1.591	.000	4.000	.007	.027	.006	−.027	.038	.119	−.014	.007	−.056	.013	−.029
25 External faculty	.342	.951	.000	4.000	.020	.048	−.016	.044	−.031	−.028	−.026	.008	−.004	−.009	.028
26 Non-faculty	.704	1.407	.000	4.000	.060	−.011	−.008	−.005	−.082	−.024	−.192	−.045	.133	.056	.012
27 #Faculty	1.249	.643	.000	2.000	.087	.045	.019	.003	−.029	.248	−.112	−.025	−.021	.034	−.011

Variables	12	13	14	15	16	17	18	19	20	21	22	23	24	25	26
1 Degree in time															
2 Academic career															
3 Related job															
4 ln(#Pub)															
5 Wage rate															
6 Ph.D. satisfaction															
7 Age															
8 Female															
9 Fellowship															
10 Job motive															
11 Academic motive															
12 Regular Ph.D.															
13 Professional Ph.D.	−.737														
14 International Ph.D.	−.427	−.296													
15 Ph.D. in Science	.185	−.200	.006												
16 Ph.D. in Engineering	−.061	−.008	.097	−.255											
17 Ph.D. in Agriculture	.000	−.034	.046	−.121	−.146										
18 Ph.D. in Health	−.119	.243	−.156	−.293	−.354	−.169									
19 Ph.D. in Humanity	.084	−.102	.018	−.139	−.167	−.080	−.193								
20 Ph.D. in Social Sci	−.046	.002	.062	−.145	−.175	−.083	−.201	−.095							
21 Ph.D. in Others	−.002	.023	−.027	−.118	−.143	−.068	−.164	−.078	−.081						
22 Univ tier	.176	−.173	−.017	.157	.094	.045	−.153	−.037	−.073	−.036					
23 Official supervisor	.070	−.132	.079	.057	.096	.023	−.097	−.060	−.008	−.023	−.038				
24 Internal faculty	−.013	−.014	.038	−.064	−.051	−.023	.132	−.027	.005	−.010	−.130	.065			
25 External faculty	.058	−.007	−.073	.040	−.037	−.030	.009	.028	−.013	.001	.018	−.170	−.293		
26 Non-faculty	.127	−.158	.032	.130	−.011	.061	−.012	−.067	−.094	−.037	.177	−.015	−.401	−.101	
27 #Faculty	.059	−.091	.038	.011	.003	−.013	.073	−.075	−.031	−.023	−.094	.499	.631	.113	−.261

*N* = 5052

### Supervisory setting

**Fig. 1:**
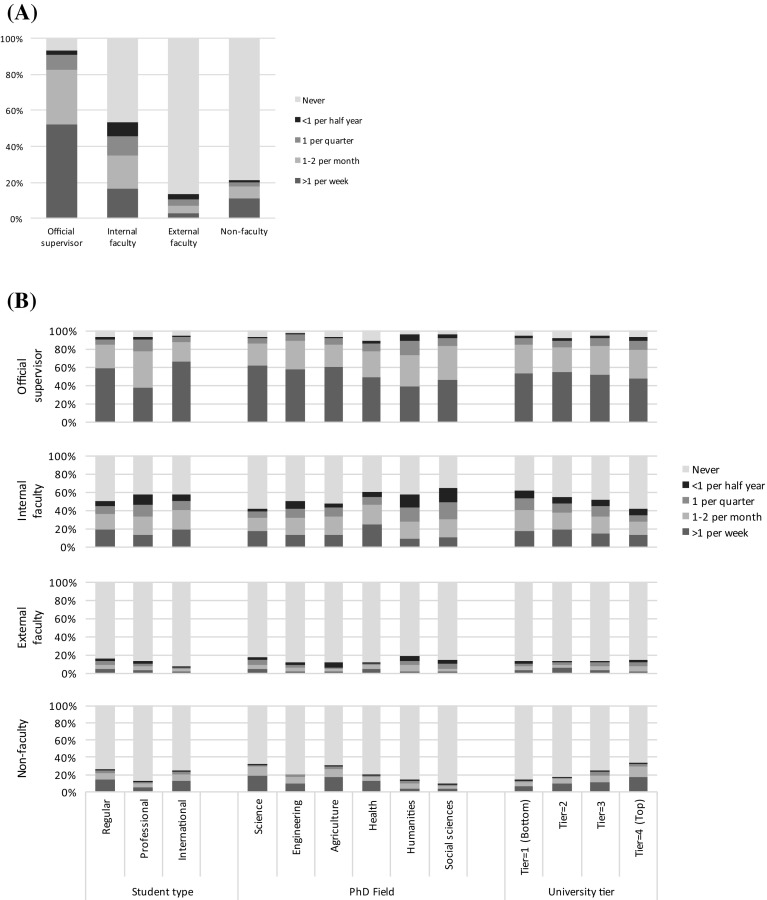
Frequency of instruction **a** frequency by instructor category, **b** breakdown

As expected, the majority of the Ph.D.s were mainly instructed by their official supervisors while some were given instruction mainly by other faculty or non-faculty members. About half of the Ph.D.s were given secondary instruction by internal faculty members. The frequency of instruction varies considerably; while 60% of Ph.D.s received weekly or more frequent instruction, 10% did so quarterly or less. Figure 1a illustrates the instruction frequency given by each instructor category: 52% of Ph.D.s received instruction from their official supervisors weekly or more frequently; 35% were instructed by internal faculty members at least monthly; 13% received any instruction by external faculty members and 21% by non-faculty researchers. Overall, 52% of Ph.D.s received frequent instruction—once a month or more frequent—by a single faculty member (i.e., one of the official supervisor, internal faculty member, or external faculty member); 36% received frequent supervision from two of them, and 11% received no frequent supervision from faculty members.

Figure 1b provides breakdowns by student types, Ph.D. fields, and university tiers. To analyze the determinants of the supervisory settings statistically, Table 2 regress the supervisory settings on several contextual variables. Since the dependent variables are all ordinal, we draw on ordinal logistic regressions. Both descriptive and regression analyses indicate some noticeable patterns in the supervisory setting. Among student types, professional Ph.D.s were least frequently instructed by official supervisors, presumably because they are in frequently present at the lab, and they were also least frequently instructed by non-faculty researchers probably due to their social status. International Ph.D.s were most often instructed by official supervisors and internal faculty members but least by external faculty members, perhaps because their network outside their main affiliation is limited. Among Ph.D. fields, Ph.D.s in Health in frequently received instruction by official supervisors but frequently by internal faculty members. This may be because of the hierarchical chair system typical in the field, where the official supervisor delegates Ph.D. supervision to junior lab members. Instruction by official supervisors is in frequent also in HASS, perhaps due to a less team-based nature of research activities in the field. Among university tiers, higher-tier universities are characterized by less frequent instruction by official supervisors but more frequent instruction by non-faculty researchers. This is probably because labs in higher-tier universities are larger and afford to use their lab members (e.g., senior students, postdocs) for Ph.D. supervision.

**Table 2:** Prediction of supervisory setting

	Model 1	Model 2	Model 3	Model 4	Model 5
	Official supervisor	Internal faculty	External faculty	Non-faculty	#Faculty
Age	−.026*** (.004)	−.002 (.004)	−.007 (.006)	−.043*** (.007)	−.019*** (.004)
Female	−.084 (.066)	−.103^†^ (.063)	.076 (.099)	−.041 (.087)	−.167* (.066)
Fellowship	−.110 (.119)	−.361** (.124)	−.129 (.174)	.416*** (.121)	−.248* (.115)
Regular Ph.D.					
Professional Ph.D.	−.509*** (.075)	−.192** (.073)	.021 (.116)	−.278* (.110)	−.356*** (.078)
International Ph.D.	.339*** (.097)	.243** (.086)	−.811*** (.170)	.115 (.113)	.117 (.090)
Ph.D. in Science					
Ph.D. in Engineering	.031 (.094)	.095 (.091)	−.436** (.135)	−.395*** (.108)	−.018 (.090)
Ph.D. in Agriculture	.019 (.136)	.154 (.129)	−.498* (.205)	.082 (.147)	−.038 (.130)
Ph.D. in Health	−.391*** (.093)	.657*** (.090)	−.430** (.132)	−.085 (.107)	.296*** (.089)
Ph.D. in Humanity	−.816*** (.119)	.139 (.115)	.146 (.161)	−.782*** (.166)	−.465*** (.124)
Ph.D. in Social Sci	−.421*** (.118)	.310** (.111)	−.165 (.171)	−.966*** (.179)	−.163 (.119)
Ph.D. in Others	−.410** (.133)	.277* (.127)	−.190 (.192)	−.493** (.179)	−.045 (.135)
Univ tier	−.189*** (.026)	−.178*** (.025)	.039 (.038)	.274*** (.033)	−.202*** (.026)
Chi-squared stat	376.292***	187.878***	58.710***	388.648***	199.787***
Log likelihood	−5530.903	−6650.746	−2774.724	−3448.492	−4494.770
*N*	4802	4809	4817	4814	4817

Unstandardized coefficients (standard errors in parentheses). Two-tailed test. ^†^ *p* < .10;* *p* < .05; ** *p* < .01; *** *p* < .001. Ordinal logistic regressions. *Regular Ph.D.* and *Ph.D. in Science* are the reference groups for student types and for Ph.D. fields respectively

### Determinants of training outcome

#### Career outcome

We examine the effect of the supervisory settings on the possibility of earning a degree in time (Table 3). Model 1 shows that frequent supervision by the official supervisor and by external faculty members is significantly positively associated with timely degree attainment. Model 2, instead, uses *#Faculty* as the main independent variable. To distinguish the impact of having a single instructor and that of having a second instructor, Model 2 includes two dummy variables with #Faculty = 1 as the reference group. The result shows that a lack of professional supervision is associated with failing to earn degrees in time, and that having multiple instructors is associated with timely degree attainment. It is plausible that supervisors decided to give frequent instructions to Ph.D.s who seemed likely to earn degrees, so Models 3 and 4 control for Ph.D.s’ motives to pursue degrees. Even after controlling for these motives, the effect of the supervisory settings remains significant, implying that frequent supervision does facilitate degree attainment. We also ran the same regression models for several sets of subsamples, finding that the effect of the supervisory settings is rather consistent between student types, Ph.D. fields, and university tiers. As to the control variables, the result suggests that young Ph.D.s, male Ph.D.s, and Ph.D.s with fellowship are more likely to earn degrees in time than otherwise.

**Table 3:** Prediction of degree awarded in time

	Model 1	Model 2	Model 3	Model 4
Age	−.044*** (.005)	−.044*** (.005)	−.044*** (.005)	−.044*** (.005)
Female	−.235** (.076)	−.232** (.075)	−.237** (.076)	−.235** (.076)
Fellowship	.951*** (.147)	.937*** (.146)	.945*** (.147)	.932*** (.147)
Regular Ph.D.				
Professional Ph.D.	.096 (.089)	.093 (.089)	.109 (.090)	.112 (.090)
International Ph.D.	.114 (.105)	.111 (.104)	.108 (.106)	.098 (.105)
Ph.D. in Science				
Ph.D. in Engineering	.574*** (.100)	.589*** (.099)	.578*** (.100)	.590*** (.100)
Ph.D. in Agriculture	.454** (.141)	.428** (.140)	.457** (.141)	.433** (.141)
Ph.D. in Health	.751*** (.099)	.714*** (.098)	.753*** (.099)	.712*** (.098)
Ph.D. in Humanity	−2.141*** (.191)	−2.121*** (.190)	−2.148*** (.191)	−2.129*** (.191)
Ph.D. in Social Sci	−1.002*** (.141)	−.974*** (.140)	−1.029*** (.142)	−1.003*** (.141)
Ph.D. in Others	−.613*** (.151)	−.618*** (.151)	−.619*** (.152)	−.627*** (.152)
Univ tier	.064* (.030)	.060* (.029)	.065* (.030)	.060* (.029)
Job motive			−.023 (.164)	−.033 (.163)
Academic motive			.065 (.070)	.078 (.070)
Official supervisor	.137*** (.029)		.137*** (.029)	
Internal faculty	.005 (.024)		.008 (.024)	
External faculty	.091* (.037)		.092* (.037)	
Non-faculty	−.027 (.026)		−.024 (.026)	
#Faculty = 0		−.297** (.108)		−.322** (.109)
#Faculty = 1				
#Faculty = 2		.116^†^ (.069)		.119^†^ (.069)
Chi-squared stat	936.802***	924.870***	939.337***	926.904***
Log likelihood	−2857.935	−2875.557	−2851.121	−2865.621
*N*	4800	4817	4792	4804

Unstandardized coefficients (standard errors in parentheses). Two-tailed test. ^†^ *p* < .10; * *p* < .05; ** *p* < .01; *** *p* < .001. Logistic regressions. *Regular Ph.D.*, *Ph.D. in Science*, and *#Faculty* = *1* are the reference groups for respective sets of independent variables

Second, Table 4 examines how the supervisory settings influence Ph.D.’s choice between academic and non-academic careers. As the dependent variable, *academic career*, is dichotomous, we use logistic regressions. Table 4A suggests that instructions by official supervisors and by external faculty members are positively associated (Model 1)—or lack of it is negatively associated (Model 2)—with academic career choice. Because Ph.D. degrees are often a precondition to obtain academic jobs, Models 3 and 4 focus on a subsample of Ph.D.s who graduated with a degree. The result indicates a similar pattern, but the supervision by the official supervisor (Model 3) as well as no frequent supervision by faculty members (Model 4) turn insignificant, suggesting that these factors affect degree attainment through which to influence the career choice indirectly. Next, since the career choice should be influenced by Ph.D.’s motives, Models 5 and 6 use a subsample of Ph.D.s who had intended to pursue academic careers from the beginning (*academic motive* = 1). The result indicates a similar pattern, suggesting that the supervisory settings do influence the career choice, although the effect of external faculty members turns insignificant. Among the control variables, the result finds that females are more likely to pursue academic careers than males. Professional Ph.D.s, who had jobs, are less likely to pursue academic careers than regular Ph.D.s because many of them continued their original jobs. On the other hand, international Ph.D.s are more likely to pursue academic careers as many of them explicitly aimed at degrees for academic career development.

**Table 4:** Prediction of academic career choice

(A) Base model						
	Model 1	Model 2	Model 3	Model 4	Model 5	Model 6
	All	All	Degree awarded	Degree awarded	Academic motive	Academic motive
Age	−.021*** (.005)	−.020*** (.005)	−.018** (.006)	−.017** (.006)	−.006 (.010)	−.005 (.010)
Female	.325*** (.074)	.336*** (.074)	.332*** (.088)	.347*** (.088)	.364** (.137)	.358** (.136)
Fellowship	.213 (.136)	.184 (.135)	.172 (.147)	.132 (.146)	.184 (.214)	.164 (.213)
Regular Ph.D.						
Professional Ph.D.	−.313*** (.086)	−.308*** (.085)	−.544*** (.100)	−.524*** (.100)	−.468** (.180)	−.482** (.179)
International Ph.D.	.280** (.106)	.258* (.105)	.339** (.122)	.320** (.121)	.095 (.160)	.072 (.160)
Ph.D. in Science						
Ph.D. in Engineering	−.335*** (.101)	−.323** (.101)	−.289** (.111)	−.277* (.110)	−.188 (.181)	−.174 (.181)
Ph.D. in Agriculture	.095 (.144)	.069 (.143)	.155 (.160)	.128 (.159)	.063 (.268)	.056 (.267)
Ph.D. in Health	.540*** (.101)	.536*** (.100)	.605*** (.113)	.599*** (.112)	.503* (.199)	.489* (.198)
Ph.D. in Humanity	.100 (.137)	.135 (.136)	.416* (.202)	.460* (.201)	−.574** (.207)	−.511* (.207)
Ph.D. in Social Sci	.205 (.135)	.232^†^ (.134)	.313^†^ (.173)	.330^†^ (.172)	.055 (.217)	.093 (.215)
Ph.D. in Others	.568*** (.157)	.583*** (.157)	.779*** (.207)	.788*** (.207)	.104 (.240)	.130 (.240)
Univ tier	.058* (.029)	.052^†^ (.029)	.066^†^ (.034)	.057^†^ (.034)	.034 (.051)	.024 (.050)
Job motive	−.499** (.160)	−.509** (.160)	−.557** (.184)	−.568** (.184)	−.609* (.291)	−.640* (.290)
Academic motive	1.119*** (.069)	1.125*** (.069)	1.228*** (.081)	1.233*** (.081)		
Official supervisor	.059* (.028)		.032 (.034)		.112* (.051)	
Internal faculty	.032 (.024)		.019 (.027)		.026 (.044)	
External faculty	.114** (.036)		.098* (.042)		.101 (.066)	
Non-faculty	−.045^†^ (.026)		−.070* (.030)		−.050 (.048)	
#Faculty = 0		−.222* (.104)		−.150 (.129)		−.463** (.174)
#Faculty = 1						
#Faculty = 2		.078 (.068)		.072 (.077)		−.010 (.123)
Chi-squared stat	634.000***	620.651***	596.708***	583.445***	62.033***	59.943***
Log likelihood	−2957.850	−2972.398	−2234.548	−2247.034	−969.812	−973.106
*N*	4792	4804	3733	3742	1772	1776

(B) Field breakdown						
	Model 1	Model 2	Model 3	Model 4	Model 5	Model 6
	STEM	STEM	Health	Health	HASS	HASS
Official supervisor	.072 (.047)		.067 (.047)		.021 (.071)	
Internal faculty	−.051 (.036)		.120** (.041)		.044 (.064)	
External faculty	.026 (.056)		.249*** (.065)		.011 (.093)	
Non-faculty	−.132*** (.037)		.061 (.047)		−.034 (.084)	
#Faculty = 0		−.116 (.174)		−.197 (.197)		−.484* (.204)
#Faculty = 1						
#Faculty = 2		.124 (.101)		.179 (.122)		−.235 (.171)
Chi-squared stat	439.050***	423.198***	124.745***	109.928***	75.878***	81.075***
Log likelihood	−1332.802	−1342.825	−875.494	−885.797	−525.423	−524.756
*N*	2240	2243	1408	1413	842	845

(C) Student type breakdown						
	Model 1	Model 2	Model 3	Model 4	Model 5	Model 6
	Regular	Regular	Professional	Professional	International	International
Official supervisor	.022 (.039)		.041 (.050)		.204* (.088)	
Internal faculty	−.035 (.033)		.148*** (.041)		−.074 (.072)	
External faculty	.043 (.048)		.237*** (.064)		.015 (.151)	
Non-faculty	−.096** (.034)		.027 (.053)		−.114 (.080)	
#Faculty = 0		−.262^†^ (.154)		−.070 (.161)		−.705* (.342)
#Faculty = 1						
#Faculty = 2		−.000 (.092)		.295* (.121)		−.182 (.197)
Chi-squared stat	266.513***	257.904***	301.583***	288.166***	56.308***	54.377***
Log likelihood	−1571.827	−1576.625	−993.665	−1004.672	−338.799	−343.201
*N*	2550	2551	1656	1662	586	591

Unstandardized coefficients (standard errors in parentheses). Two-tailed test. ^†^ *p* < .10; * *p* < .05; ** *p* < .01; *** *p* < .001. Logistic regressions. *Regular Ph.D.*, *Ph.D. in Science*, and *#Faculty* = *1* are the reference groups for respective sets of independent variables. In Tables (B) and (C), the control variables are omitted for parsimony

Since Table 4A indicates significant differences between Ph.D. fields, Table 4B splits the sample by Ph.D. fields into STEM (Science, Engineering, and Agriculture), Health, and HASS (Humanities and Social sciences). In STEM, non-faculty’s supervision shows a significantly negative effect (Model 1). Frequent instruction by non-faculty researchers, presumably senior students and postdocs in the same lab, might imply that the lab was large and internal competition was severe, and thus, Ph.D.s might find it difficult to pursue academic careers. In HASS, on the other hand, a lack of faculty’s supervision shows a significantly negative effect (Model 6). This is perhaps because the less team-based nature of HASS research makes an instruction by a single faculty member all the more influential.

Similarly, Table 4C breaks down student types, presenting clear differences. For international students, connection with the official supervisor is indispensable due to their limited local network (Ch. 5.1). Thus, instruction by the official supervisor (Model 5) or lack of it (Model 6) has significant impact. In contrast, professional students could have broader network beyond their official supervisors, and successfully exploiting it increases the likelihood of choosing academic careers after graduation (Models 3 and 4). For regular students, instruction by non-faculty members (Model 1) or lack of instruction by faculty members (Model 2) discourages academic career choice.

As the third measure of career outcomes, Table 5 examines how areas of jobs can be influenced by supervisory settings. Model 1 shows that the instruction by official supervisors is positively associated with job relatedness, implying that frequent instruction by supervisors reinforces Ph.D.s’ interest and encourages them to continue research in the same field. The model finds that *academic career* has a significantly positive effect because Ph.D.s at academic jobs are likely to continue related jobs. Thus, we split Ph.D.s who chose academic jobs (Models 3 and 4) and Ph.D.s who chose non-academic jobs (Models 5 and 6), to find that the effect of supervisory settings is significant only for the academic subsample.

**Table 5:** Prediction of job relatedness

	Model 1	Model 2	Model 3	Model 4	Model 5	Model 6
	All	All	Academic	Academic	Non-academic	Non-academic
Age	−.009 (.007)	−.010 (.007)	−.015 (.013)	−.012 (.013)	−.009 (.009)	−.011 (.009)
Female	−.320** (.117)	−.316** (.117)	−.081 (.198)	−.019 (.197)	−.426** (.149)	−.421** (.148)
Fellowship	.441^†^ (.248)	.469^†^ (.247)	1.204* (.604)	1.208* (.603)	.232 (.287)	.282 (.285)
Regular Ph.D.						
Professional Ph.D.	.208 (.140)	.225 (.139)	−.477* (.243)	−.483* (.241)	.520** (.172)	.521** (.170)
International Ph.D.	.205 (.182)	.203 (.180)	.738* (.368)	.756* (.367)	−.050 (.225)	−.065 (.222)
Ph.D. in Science						
Ph.D. in Engineering	.423** (.157)	.455** (.155)	.450 (.336)	.496 (.335)	.405* (.181)	.414* (.179)
Ph.D. in Agriculture	.154 (.219)	.134 (.217)	−.497 (.347)	−.542 (.347)	.567* (.287)	.538^†^ (.282)
Ph.D. in Health	.375* (.156)	.336* (.154)	.612* (.292)	.457 (.288)	.367^†^ (.189)	.364^†^ (.187)
Ph.D. in Humanity	.151 (.220)	.120 (.220)	.333 (.374)	.236 (.372)	.182 (.286)	.144 (.285)
Ph.D. in Social Sci	.061 (.208)	.077 (.207)	.294 (.374)	.277 (.371)	−.033 (.263)	−.024 (.261)
Ph.D. in Others	.416 (.253)	.400 (.252)	.731^†^ (.438)	.646 (.436)	.317 (.323)	.297 (.321)
Univ tier	.081^†^ (.046)	.072 (.046)	.173* (.085)	.153^†^ (.084)	.028 (.056)	.026 (.056)
Job motive	−.808*** (.211)	−.799*** (.210)	−.571 (.446)	−.552 (.446)	−.829*** (.249)	−.809** (.248)
Academic motive	−.076 (.117)	−.077 (.117)	.190 (.187)	.214 (.186)	−.270^†^ (.150)	−.270^†^ (.149)
Academic career	1.586*** (.115)	1.593*** (.114)				
Official supervisor	.100* (.043)		.204** (.072)		.042 (.053)	
Internal faculty	−.018 (.037)		−.043 (.066)		.021 (.046)	
External faculty	−.078 (.054)		−.001 (.097)		−.099 (.067)	
Non-faculty	−.019 (.041)		−.110 (.073)		.041 (.050)	
#Faculty = 0		.163 (.165)		.416 (.322)		.076 (.195)
#Faculty = 1						
#Faculty = 2		.173 (.110)		.462* (.201)		.089 (.134)
Chi-squared stat	284.524***	279.759***	57.227***	52.244***	61.827***	56.832***
Log likelihood	−1413.330	−1419.080	−511.399	−514.300	−869.655	−874.228
*N*	4559	4570	2721	2729	1838	1841

Unstandardized coefficients (standard errors in parentheses). Two-tailed test. ^†^ *p* < .10; * *p* < .05; ** *p* < .01; *** *p* < .001. Logistic regressions. *Regular Ph.D.*, *Ph.D. in Science*, and *#Faculty* = *1* are the reference groups for respective sets of independent variables

Models 5 and 6 also show that Ph.D.s in Engineering, Agriculture, and Health tend to engage in related jobs in industry. As these three fields are applied, this result might suggest that these fields are successfully transferring knowledge workers to industry, as designed. Interestingly, the models show that Ph.D.s who intended to delay job hunting are likely to find jobs unrelated to Ph.D. subjects. Thus, training for Ph.D.s with such a motive may be ineffective in transferring knowledge workers to industry.

#### Performance

Next, we examine the impact of supervisory settings on Ph.D.s’ performance drawing on two measurements. First, we use the publication count as the measure of scientific performance (Table 6). Since this is a count variable, we use negative binomial regressions. Model 1 shows that instruction by external faculty members is positively associated with the publication count, suggesting that an external information source has positive impact on scientific performance. We suspect that the contribution of supervision should differ by the scientific performance of the instructors, and thus, the sample is split into high-tier and low-tier university subsamples (Models 3–6). Indeed, Model 3 indicates that instruction by external faculty members is positively associated with scientific performance only in high-tier universities. Interestingly, Model 4 shows negative coefficients for instructions by the official supervisor and by internal faculty members. This is probably due to a reverse causality; that is, poorly performing Ph.D.s in low-tier universities needed frequent supervision. Concerning control variables, Table 6 shows that female Ph.D.s publish less than male Ph.D.s. Fellowship is associated with more publications. Professional Ph.D.s publish more than regular Ph.D.s, perhaps because they have longer academic careers before enrolling in Ph.D. programs. International Ph.D.s also perform better than regular Ph.D.s. Academic motive shows significantly positive coefficients and job motive negative coefficients, suggesting that scientific performance is predictable to some extent by their initial motives for Ph.D. degrees.

**Table 6:** Prediction of publication performance

	Model 1	Model 2	Model 3	Model 4	Model 5	Model 6
	All	All	High univ tier	Low univ tier	High univ tier	Low univ tier
Age	.001 (.002)	.002 (.002)	−.003 (.004)	.003 (.003)	−.003 (.004)	.004 (.003)
Female	−.110** (.034)	−.109** (.034)	−.185*** (.050)	−.062 (.046)	−.184*** (.050)	−.063 (.046)
Fellowship	.210*** (.052)	.204*** (.052)	.207*** (.057)	.212^†^ (.119)	.200*** (.057)	.202^†^ (.119)
Regular Ph.D.						
Professional Ph.D.	.212*** (.041)	.218*** (.041)	.214*** (.065)	.232*** (.054)	.212** (.065)	.239*** (.054)
International Ph.D.	.261*** (.044)	.243*** (.044)	.243*** (.061)	.279*** (.063)	.227*** (.061)	.258*** (.062)
Ph.D. in Science						
Ph.D. in Engineering	.172*** (.045)	.170*** (.045)	.219*** (.056)	.105 (.074)	.216*** (.056)	.105 (.074)
Ph.D. in Agriculture	.196** (.065)	.185** (.065)	.135 (.085)	.265** (.099)	.123 (.085)	.259** (.099)
Ph.D. in Health	.105* (.046)	.112* (.046)	.106^†^ (.063)	.081 (.071)	.112 ^†^ (.063)	.088 (.070)
Ph.D. in Humanity	.092 (.059)	.108^†^ (.059)	.146^†^ (.081)	.052 (.088)	.162* (.081)	.063 (.088)
Ph.D. in Social Sci	−.104^†^ (.060)	−.105^†^ (.060)	−.068 (.084)	−.144^†^ (.088)	−.069 (.084)	−.150^†^ (.087)
Ph.D. in Others	.099 (.065)	.102 (.065)	.328*** (.086)	−.133 (.098)	.324*** (.086)	−.123 (.098)
Univ tier	.033* (.013)	.033** (.013)				
Job motive	−.170* (.080)	−.175* (.080)	−.273** (.099)	−.029 (.132)	−.280** (.099)	−.029 (.133)
Academic motive	.148*** (.029)	.152*** (.029)	.090* (.040)	.205*** (.042)	.091* (.040)	.207*** (.042)
Official supervisor	−.020 (.013)		−.002 (.018)	−.038* (.019)		
Internal faculty	.000 (.011)		.025 (.015)	−.025^†^ (.015)		
External faculty	.039* (.016)		.059** (.022)	.021 (.022)		
Non-faculty	−.010 (.012)		−.003 (.016)	−.009 (.019)		
#Faculty = 0		.008 (.045)			−.026 (.061)	.048 (.067)
#Faculty = 1						
#Faculty = 2		.003 (.030)			.054 (.043)	−.056 (.043)
Chi-squared stat	167.356***	154.445***	98.615***	101.888***	91.142***	92.576***
Log likelihood	−8294.305	−8318.113	−3959.089	−4312.664	−3968.817	−4328.656
*N*	3563	3572	1701	1862	1704	1868

Unstandardized coefficients (standard errors in parentheses). Two-tailed test. ^†^ *p* < .10; * *p* < .05; ** *p* < .01; *** *p* < .001. Negative binomial regressions. *Regular Ph.D.*, *Ph.D. in Science*, and *#Faculty* = *1* are the reference groups for respective sets of independent variables. High univ tier: *Univ tier* = 3 or 4, Low univ tier: *Univ tier* = 1 or 2

Since publication performance may not be an ideal measure of non-academic performance, we also draw on the wage rate as a proxy of performance (Table 7). From the analysis, we exclude Ph.D.s who are employed in academic because their salary is usually set by a formula and non-negotiable. Though Models 1 and 2 found no significant effect of supervisory settings, subsample analyses splitting university tiers suggest that instruction by official supervisors and instruction by multiple faculty members are effective only in high-tier universities. As for control variables, age has a significantly positive effect because the salary system in Japan is often seniority-based. Females earn less than males. Professional Ph.D.s earn more than regular Ph.D.s for their supposedly higher skills and longer professional experience. International Ph.D.s earn less than regular Ph.D.s, even though the former exceeds the latter in publication performance. This is partly because the majority of international Ph.D.s found jobs outside Japan, where the salary standard is lower. Academic motive is negatively associated with the wage rate in non-academia, suggesting that those who initially intended to pursue academic careers but ended in non-academic careers earn less than those who did not have such initial intention.

**Table 7:** Prediction of wage rate for non-academic Ph.D. sample

	Model 1	Model 2	Model 3	Model 4	Model 5	Model 6
	All	All	High univ tier	Low univ tier	High univ tier	Low univ tier
Age	.057*** (.005)	.058*** (.005)	.051*** (.007)	.060*** (.007)	.051*** (.007)	.060*** (.007)
Female	−.313*** (.088)	−.306*** (.088)	−.366** (.129)	−.297* (.120)	−.360** (.129)	−.287* (.119)
Fellowship	−.044 (.155)	−.038 (.155)	−.058 (.148)	.233 (.415)	−.052 (.147)	.239 (.412)
Regular Ph.D.						
Professional Ph.D.	.633*** (.092)	.628*** (.091)	.740*** (.130)	.582*** (.129)	.727*** (.127)	.573*** (.128)
International Ph.D.	−.482*** (.137)	−.478*** (.137)	−.329^†^ (.171)	−.608** (.209)	−.328^†^ (.170)	−.604** (.208)
Ph.D. in Science						
Ph.D. in Engineering	.062 (.102)	.064 (.102)	.108 (.120)	−.016 (.165)	.119 (.119)	−.023 (.164)
Ph.D. in Agriculture	−.331* (.152)	−.331* (.152)	−.048 (.188)	−.587* (.235)	−.031 (.187)	−.592* (.235)
Ph.D. in Health	.767*** (.107)	.774*** (.106)	.883*** (.138)	.665*** (.161)	.898*** (.136)	.667*** (.160)
Ph.D. in Humanity	−.824*** (.174)	−.836*** (.174)	−.733** (.235)	−.939*** (.251)	−.773*** (.234)	−.959*** (.250)
Ph.D. in Social Sci	−.153 (.158)	−.146 (.157)	−.048 (.226)	−.225 (.222)	−.024 (.224)	−.236 (.221)
Ph.D. in Others	−.330^†^ (.186)	−.331^†^ (.186)	−.494^†^ (.282)	−.324 (.254)	−.500^†^ (.280)	−.325 (.253)
Univ tier	.062* (.031)	.064* (.031)				
Job motive	−.150 (.161)	−.153 (.161)	−.143 (.185)	−.145 (.265)	−.152 (.184)	−.140 (.265)
Academic motive	−.261** (.087)	−.272** (.087)	−.170 (.110)	−.360** (.132)	−.176 (.110)	−.380** (.132)
Official supervisor	−.015 (.030)		.039 (.039)	−.056 (.045)		
Internal faculty	.034 (.025)		.077* (.034)	.006 (.036)		
External faculty	−.024 (.040)		−.018 (.053)	−.030 (.059)		
Non-faculty	.004 (.028)		.011 (.034)	.006 (.043)		
#Faculty = 0		.055 (.106)			−.016 (.135)	.131 (.157)
#Faculty = 1						
#Faculty = 2		.120 (.074)			.281** (.097)	.009 (.106)
F Test	47.837***	53.639***	27.938***	25.477***	31.805***	28.734***
Adjusted R-squared	.319	.318	.370	.290	.372	.289
*N*	1803	1805	781	1022	781	1024

Unstandardized coefficients (standard errors in parentheses). Two-tailed test. ^†^ *p* < .10; * *p* < .05; ** *p* < .01; *** *p* < .001. Ordinary least squares. *Regular Ph.D.*, *Ph.D. in Science*, and *#Faculty* = *1* are the reference groups for respective sets of independent variables. High univ tier: *Univ tier* = 3 or 4, Low univ tier: *Univ tier* = 1 or 2

#### Ph.D. satisfaction

Finally, Table 8 predicts respondents’ satisfaction with Ph.D. programs. As the dependent variable is ordinal, we use ordinal logistic regressions. Both Models 1 and 2 show that frequent supervision significantly increases the degree of satisfaction. Unlike in the previous sections, instruction even by non-academic researchers contributes to Ph.D.s’ satisfaction. The effect is almost universal across Ph.D. fields and university tiers.

**Table 8:** Prediction of Ph.D.’s satisfaction

	Model 1	Model 2
Age	.016*** (.004)	.011** (.004)
Female	−.023 (.065)	−.042 (.064)
Fellowship	.282* (.116)	.255* (.115)
Regular Ph.D.		
Professional Ph.D.	.459*** (.077)	.354*** (.076)
International Ph.D.	.589*** (.093)	.599*** (.092)
Ph.D. in Science		
Ph.D. in Engineering	.262** (.091)	.262** (.090)
Ph.D. in Agriculture	−.133 (.127)	−.108 (.126)
Ph.D. in Health	−.249** (.090)	−.325*** (.088)
Ph.D. in Humanity	.176 (.120)	.124 (.119)
Ph.D. in Social Sci	.176 (.120)	.125 (.118)
Ph.D. in Others	.101 (.133)	.051 (.132)
Univ tier	.026 (.025)	.011 (.025)
Job motive	−.495*** (.140)	−.460** (.140)
Academic motive	−.088 (.062)	−.078 (.061)
Academic career	.196*** (.060)	.192** (.059)
Official supervisor	.574*** (.027)	
Internal faculty	.220*** (.021)	
External faculty	.207*** (.032)	
Non-faculty	.136*** (.023)	
#Faculty = 0		−1.297*** (.092)
#Faculty = 1		
#Faculty = 2		.455*** (.060)
Chi-squared stat	707.569***	466.210***
Log likelihood	−5648.357	−5784.722
*N*	4787	4798

Unstandardized coefficients (standard errors in parentheses). Two-tailed test. ^†^ *p* < .10; * *p* < .05; ** *p* < .01; *** *p* < .001. Ordinal logistic regressions. *Regular Ph.D.*, *Ph.D. in Science*, and *#Faculty* = *1* are the reference groups for respective sets of independent variables

## Discussions

As the modern society is increasingly becoming knowledge-driven, high-skilled knowledge workers are crucial for the sustainable development of the society (Bozeman et al. 2001). Although postgraduate education is pivotal in this regard, it has not necessarily been successful in producing human capital that meets the societal needs (Cyranoski et al. 2011; Gould 2015). Issues in academic training are attributable to gaps both in policy practices and in theories between higher education and scientific production, but empirical limitations are also responsible. That is, poor access to the inside of academic labs along with difficulty in identifying early careers of Ph.D. graduates have been undermining our understanding of academic training. To fill in these gaps, the current study aims to illustrate Ph.D. supervisory settings and investigate their impact on several outcome aspects, drawing on the national survey of a cohort of 5000 Ph.D. graduates from Japanese universities.

The result first shows that most Ph.D.s received instructions by their official supervisors, and that half of them received additional instruction by internal faculty members. The frequency of instruction has substantial variation; the majority of Ph.D.s received weekly instruction but some less than quarterly. Some Ph.D.s received instruction by non-faculty members, such as senior students and postdocs. Overall, a great deal of variation is observed in the Ph.D. supervisory setting both in quantity and in quality.

We find that these variations produce significant differences in training outcome. In terms of career outcome, the result first suggests that frequent instruction by faculty members (but not by non-faculty researchers) increases the likelihood of earning degrees in time, which is consistent with Wright and Lodwick (1989). The result also suggests that frequent supervision and supervision by multiple instructors increases the possibility of finding jobs related to dissertation subjects. Finally, the result suggests that frequent supervision by faculty members increases the likelihood of choosing academic careers whereas that by non-faculty members decreases it. Overall, the intensity of supervision seems to facilitate Ph.D.’s learning and motivations to continue related jobs in the same sector and field. The training effect differs by the type of instructors. Noticeably, non-faculty members’ instruction leads to non-academic career choice, perhaps because lack of professional instruction discourages Ph.D.s from pursuing academic careers.

These results offer a few policy implications. Successfully earning degrees is obviously desirable and finding jobs related to Ph.D. research subjects also seems efficient. In this regard, recent policies in Japan and some other countries might have created an undesirable situation in that they have allowed over-concentration of Ph.D.s in a small number of labs, where supervisors can spare insufficient time for the instruction of each Ph.D. student (Shibayama and Baba 2015). Indeed, our result shows that instruction by faculty members is significantly less frequent in higher-tier universities. Therefore, it is advisable to adequately control the number of Ph.D.s that a supervisor can actually supervise. The choice between academic and non-academic careers needs careful interpretation, since modern higher education system is expected to supply Ph.D.s to both academic and non-academic sectors. The result indicates that academic career choice is positively correlated with other outcome measures except for the wage rate, which seems to imply that unsuccessful or unsatisfied Ph.D.s opt out of academic careers. Thus, training programs for academic and non-academic careers might need to be differentiated (e.g., distinct courses, training by practitioners for the latter) (Gould 2015).

Concerning the performance outcome, the result finds that the frequent supervision by faculty members increases publication performance as well as the wage rate only in high-tier universities. Thus, training effect on performance might be contingent to supervisors’ scientific capabilities. This interpretation is consistent with Long et al. (1979). The result also points to the necessity for faculty members to allocate sufficient time and resources for training in high-tier universities, where over-capacity has been pointed out (Shibayama and Baba 2015). In addition, the result suggests that instruction by multiple supervisors increases the wage rate in industry jobs. This might suggest the importance of interdisciplinary or diverse perspectives particularly when Ph.D.s choose to work in industries.

Finally, the result suggests that frequent supervision both by faculty members and by non-faculty researchers increases Ph.D.s’ satisfaction. This is consistent with previous findings in educational psychology (Brown and Atkins 1988; Hockey 1991). It is noteworthy that Ph.D.’s satisfaction is the only outcome positively associated with instruction by non-faculty researchers. Thus, Ph.D.s can be satisfied even when their performance is not improved. In this regard, Ph.D.s’ subjective evaluation needs cautious interpretation if it is used for policymaking purposes.

These results warrant some reservations, and future research is needed to further our understanding in academic training. The sample specificity restricts the generalizability of the findings, as postgraduate education systems considerably differ by country. The outcome measures can be improved. In particular, scientific performance and the wage rate are measured 1.5 years after graduation. As the effect of academic training might take time to realize, longer-term performance measures could offer clearer results. In addition, as a career outcome, inbreeding, where students continue working under the same supervisor, is of both theoretical and practical interest (Horta et al. 2010). This is particularly so in Japan, where inbreeding is still common (Morichika and Shibayama 2015). The explanatory variables can be similarly improved. Our survey inquired into the frequency of supervision from four sources of instructors, but further details of inter-personal relationships could help interpret our results. For example, though our results mostly show desirable effects of frequent instruction by supervisors, it is plausible that excessive control hinders Ph.D.s’ creativity. Testing such hypotheses takes more detailed information such as training styles (Hockey 1996) and research task allocation (Kam 1997). The expertise fields of instructors can be informative in evaluating the diversity of supervising teams (Spelt et al. 2009). Since a lab is a complex organization, the effect of training and the effect of other factors need to be disentangled. Further data on organizational settings, such as lab size and age, can be of use in this regard (e.g., Heinze et al. 2009; Pelz and Andrews 1966). In regression analyses, endogeneity is concerned. In particular, supervisors might have decided the frequency of training and other training styles on the basis of Ph.D.’s latent capabilities. We plan to conduct follow-up surveys of the same cohort of Ph.D.s, which we expect can address a part of these issues.

## Footnotes

1 Source: School Basic Survey (http://www.mext.go.jp/b_menu/toukei/chousa01/kihon/1267995.htm).

2 A few universities offer 5-year integrated Ph.D. programs. Ph.D. programs in some fields take four years, such as in Medicine, Veterinary sciences, and Pharmacy.

3 Students can stay in a Ph.D. program up to a certain number of years (typically double the standard number of years). Apart from this rule, most Ph.D. programs do not have a mechanism to force out students. Since students can graduate without earning a degree, drop out, where students leave the program without graduation, is rather rare. Graduation without a degree allows students to save tuition fees compared to staying in the program for a long period. It is possible to earn a degree many years after graduation.

4 Until a reform in 2005, the government controlled the admission quota of postgraduate courses.

5 In STEM fields, a Ph.D. degree is almost a requirement for professional academic careers currently. Ph.D. graduates typically experience several years of a postdoc period before earning junior faculty positions. For example, 44% of Science Ph.D. graduates in 2002–2006 became postdocs while only 6.2% obtained faculty positions immediately after graduation (Misu et al. 2010).

6 Note that the survey inquired into only the first and the second instructors. For the first set, the survey ignores third and fourth instructors, if any. We assume that their instruction frequency was negligible and coded the variables 0 if the category was not included in the first and second instructors. The second-set variable is right-censored. In addition, it overlooks the possibility that a student is supervised by, for example, two internal faculty members. In this regard, precisely speaking, the variable may be associated with the diversity of supervisors rather than their number.

7 4.5% of the respondents were not employed.

8 The publication share of each university among all publications with Japanese addresses: >5% (tier = 4), 1–5% (tier = 3), .5–1% (tier = 2), and <.5% (tier = 1).

9 The national government offers a fellowship for three years. The selection is based on the applicant's performance before Ph.D. (i.e., mostly during the master program).
